# Sexual behavior among volunteers enrolled in a Phase I HIV vaccine trial: experience of Projet San Francisco in Kigali, Rwanda

**DOI:** 10.1186/1742-4690-9-S2-P116

**Published:** 2012-09-13

**Authors:** J Mukamuyango, E Karita, R Bayingana, J Nyombayire, R Ingabire, S Allen, A Tichacek, P Fast, M Price, C Schmidt

**Affiliations:** 1 Projet San Francisco, Kigali, Rwanda; 2 Emory University, Atlanta, GA, USA; 3 International AIDS Vaccine Initiative, New York, NY, USA

## Background

Phase 1 HIV vaccine trials are conducted among HIV-uninfected, healthy volunteers at low risk for HIV. Study volunteers are counseled to maintain low risk behavior for HIV acquisition. The objective of this study was to assess sexual behavior of volunteers in a Phase 1 vaccine trial, conducted in Kigali, Rwanda.

## Methods

Concordant HIV-negative couples who were counseled and tested together were considered as low risk group for HIV acquisition and were invited to participate in Phase 1 HIV vaccine trial to evaluate safety and immunogenicity of a multiclade HIV-1 DNA plasmid vaccine followed by a recombinant, multiclade HIV-1 adenoviral vector vaccine (IAVI V001). HIV risk reduction counseling, assessment of sexual behavior and screening for sexually transmitted infections (STI) were conducted quarterly. After completion of the vaccine trial, participants and their partners were invited to participate in a long-term 5-year follow-up study from their last vaccination for further assessment of sexual behavior and continuous risk reduction counseling.

## Results

Between November 2005 and May 2006, 57 volunteers (36 men and 21 women) were enrolled in the clinical trial. All volunteers completed their trial visits. During the first twelve months following the first vaccination, none of the study participants reported sex with other partners nor was treated for STI. After unblinding, 55 volunteers agreed to continue in long-term follow up study. Through January 2012 6 men and 2 women reported having sex at least once with other partners; two were treated for STI, and one subsequently acquired HIV. (HIV incidence rate: 0.3/100 person-years).

## Conclusion

This trial and subsequent follow-up study confirm our previous findings that concordant HIV-negative couples are at low risk of HIV infection and suitable for enrolling in Phase 1 HIV prevention trials. Ongoing HIV risk reduction counseling should be provided during the trial as well as after the trail, if possible.

